# Admission Rate of Patients Visiting Emergency Department in a Tertiary Care Center in Kathmandu: A Descriptive Cross-sectional Study

**DOI:** 10.31729/jnma.8767

**Published:** 2024-09-30

**Authors:** Sanjeeb Tiwari, Jyostshna Sharma Tiwari, Jay Bhushan Jha, Sushant Regmi, Dhirendra Yadav, Ravi Kafle, Inesh Khanal, Aakripa Rani Shrestha, Shubham Shrestha, Yagya Laxmi Shakya, Ramesh Kumar Maharjan, Sanjay Kumar Gauta

**Affiliations:** 1 Department of General Practice, Tribhuwan University Teaching Hospital, Maharajgunj, Nepal; 2 Department of Obstetrics and Gynaecology, Kathmandu Medical College andn Teaching Hospital, Sinamangal, Kathmandu, Nepal; 3 Patan Academy of Health Sciences, Lagankhel, Lalitpur, Nepal; 4 Department of Emergency Medicine, Tribhuwan University Teaching Hospital, Maharajgunj, Nepal

**Keywords:** *emergency department*, *hospital admission*, *measures*, *Nepal*

## Abstract

**Introduction:**

Emergency departments serve as the initial point of contact for patients with a wide range of conditions. Not all patients visiting get admitted to the hospital. The admission rate varies from 20% in the United States to 40.83% in Nepal. Given the variability in these results across different regions, there is a need to better understand the admission rates from the Emergency Department.

**Methods:**

A descriptive cross-sectional study was conducted after obtaining ethical approval(Reference Number: 58 (6-11)E2 081/082) in the Emergency Department of a tertiary health care center. Retrospective data was collected from the data register. The data from April 2023 to April 2024 was collected. A sample size of 222 was calculated and convenience sampling was done. Demographic details like age, sex, and data regarding admission status were collected. Data was collected in MS Excel and was analyzed using JASP software.

**Results:**

Out of 222 patients that presented to the emergency department, 53 (23.87%)(18.26-29.47,95 % Confidence Interval) were admitted to the ward or intensive care unit of the hospital, whereas 167 (75.23%) got discharged, 1 (0.45%) was brought dead and 1 (0.45%) expired. The median age of patients was 43 years (IQR: 26-62). The admission rate of females was 20 (17.24%), whereas for males it was 33 (31.13%).

**Conclusions:**

The prevalence of admission of patients visiting the emergency department was higher than similar US based studies. There was a higher percentage of female patients presenting to the emergency department, while those being admitted were mostly males.

## INTRODUCTION

Emergency departments (EDs) serve as the initial point of contact for patients with a wide range of conditions, from minor injuries to life-threatening emergencies. However, not all patients visiting the ED get admitted to the hospital for further management. In a study done in the United States (US), the admission rate of patients who presented to the ED was 20%.^1^ In a similar study conducted in the US, the admission rate of the patients from the ED was 17.5%.^2^ In another study done in Thailand, among patients visiting the ED, 40.82% were admitted to the hospital for further management.^3^ The admission rate from ED of a tertiary care center in Nepal was 40.83%.^4^

Given the variability in these results across different regions and healthcare systems, there is a need to better understand the admission rates from EDs in the context of a tertiary care center in Nepal.

This study aims to find the admission rate of patient visiting emergency department at a tertiary care center.

## METHODS

A descriptive cross-sectional study was carried out after obtaining ethical approval from the IRC (Institutional Review Committee) of Tribhuvan University Teaching Hospital (TUTH), Kathmandu (Reference number: 58 (6-11)E2 081/082). This study employed a quantitative, retrospective approach to investigate the admission patterns among patients visiting the Emergency Department of Tribhuvan University Teaching Hospital, (TUTH) a tertiary-level hospital in Kathmandu, Nepal.

A convenience sampling method was used, and the sample size was calculated using:


$$\begin{array}{ll} \text{n} & ={\text{Z}}^{2}\times \frac{\text{p}\times \text{q}}{{\text{e}}^{\text{2}}}\\ & =1.96^{2}\times \frac{0.175\times 0.825}{0.05^{2}}\\ & =222 \end{array}$$

where,

n = minimum required sample size
z = 1.96 at 95% Confidence Interval (CI)
p = prevalence of admission rate, 17.5%^2^
q = 1 - p
e = margin of error, 5%

The minimum sample size was 222.

Patients who were admitted to the emergency department for treatment with complete records were included, while those with incomplete records were excluded. The study variables included the admission rate, demographic details (age and sex), and admission status in the ED. An ED register was used to collect the data, which was then entered into Microsoft Excel and analyzed using JASP software.

Descriptive statistics, such as frequencies, percentages, and measures of central tendency, were employed to summarise the characteristics of the study population.

## RESULTS

Among 222 patients, there were 116 (52.25%) females. The median age of patients was 43 years (IQR: 26-62). In the age group of 20-29 years, there were 51 (22.97%) patients, and 29 (13.06%) patients were in the age group of 40-49 years (Table 1).

**Table 1:** Demographic Details of Patients visiting Emergency Department (n=222).

Sex	n (%)
Female	116 (52.25)
Male	106 (47.75)
Age Category	N(%)
0-9	9 (4.05)
10-19	13 (5.86)
20-29	51 (22.97)
30-39	25 (11.26)
40-49	29 (13.06)
50-59	28 (12.61)
60-69	36 (16.22)
70-79	23 (10.36)
80-89	8 (3.6)

Out of 222 patients, that presented to ER, 53 (23.87%) were admitted to the ward or Intensive care unit (ICU) of the hospital, whereas 167 (75.23) got discharged, 1 (0.45%) was brought dead and 1 (0.45%) expired in ER (Table 2).

**Table 2:** Admission and Discharge Rate of Patient presenting to Emergency Department (n=222).

Status	n(%)
Admitted	53 (23.87)
Discharged	167 (75.23)
Brought Dead	1 (0.45)
Expired	1 (0.45)

The admission rate of female patients was 20 (17.24%), whereas for male patients it was 33 (31.13%). Among male patients, 1 (0.94%) was brought dead in the ER, and 1 (0.94%) expired in the ER (Table 3).

**Table 3:** Sex Wise Admission and Discharge Rate of Patients presenting to Emergency Department (n=222).

Sex	Status	n(%)
Female (n=116)	Admitted	20 (17.24%)
	Discharged	96 (82.76%)
Male (n=106)	Admitted	33 (31.13%)
	Discharged	71 (66.98%)
	Brought Dead	1 (0.94%)
	Expired	1 (0.94%)

While distributing the age with an interval of 10 years, the admission rate of 60-69 years was 12 (33.33%), also 1 (0.94%) was brought dead in the ER, and 1 (0.94%) expired in the ER in the same group. The admission rate of 80-89 years was 3 (37.5%) (Table 4).

**Table 4:** Admission Rate and Discharge of Different Age Groups (n=222)

Age Category	Admission Status	n (%)
0-9 (n=9)	Admitted	2 (22.22%)
	Discharged	7 (77.78%)
10-19 (n=13)	Admitted	1 (7.69%)
	Discharged	12 (92.31%)
20-29 (n=51)	Admitted	11 (21.57%)
	Discharged	40 (78.43%)
30-39 (n=25)	Admitted	4 (16%)
	Discharged	21 (84%)
40-49 (n=29)	Admitted	6 (20.69%)
	Discharged	23 (79.31%)
50-59 (n=28)	Admitted	8 (28.57%)
	Discharged	20 (71.43%)
60-69 (n=36)	Admitted	12 (33.33%)
	Discharged	22 (61.11%)
	Brought Dead	1 (2.78%)
	Expired	1 (2.78%)
70-79 (n=23)	Admitted	6 (26.09%)
	Discharged	17 (73.91%)
80-89 (n=8)	Admitted	3 (37.50%)
	Discharged	5 (62.50%)

## DISCUSSION

In this study, we found that the admission rate from the ED was 23.87%. This is higher as compared to a study conducted in the US, where the admission rate was 17.5%.^2^ This could be since this US based study was multicentric whereas ours is a single-centric study. It may have introduced selection bias, as the population and practices at our institution may differ from those at multiple centers in the US.

In another study in the US, of all ED visits, 20% resulted in admission.^1^ This is similar to our study. The inclusion criteria of this study are similar to our study. Both studies included all patients who visited the ED, providing a broad and comprehensive assessment of admission rates across diverse patient groups. This approach helps ensure that the findings are reflective of the overall population visiting the ED, rather than being limited to specific subgroups.

The median sample age was 44 years, and predominantly female, 58% in this US based study.^1^ In our study, the median age of patients was 43 years with a female preponderance too i.e. 52.25%. The close alignment in the median age and gender distribution between the two studies suggests that both populations are demographically similar, which may contribute to the comparable admission rates observed. The female preponderance could reflect gender-specific health-seeking behaviors or the prevalence of certain conditions that are more common in females, potentially influencing the likelihood of admission. This was in contrast to a systematic review which revealed male patients were likely to visit the ED.^5^ This difference could be due to the higher-risk activities, which include dangerous sports, manual labor, and occupational hazards.^6,7^ Males are also less likely to seek preventive healthcare or regular medical checkups compared to women. This leads to more severe health issues by the time they seek care, often resulting in ED visits and subsequent admissions.^8^ Certain acute health conditions, such as myocardial infarctions, are more common in men, which can lead to more frequent ED visits and admissions.^6,9^

In our study, the admission rates were highest in the age groups 80-89 years and 60-69 years, which were 37.50% and 33.33% respectively. This is corroborated by a US based systematic review which shows the highest rate of ED admission was 29.0-39.4% were aged 85 or 86 years.^5^ The term "elderly" generally refers to individuals who are older in age, typically defined as those who are 65 years and older.^10,11^ The elderly population has increased vulnerability to acute illness, suffers from multiple chronic conditions and is at a higher risk of complications.^12-16^

In a systematic review of seven studies, the patients that died in the ED ranged from 0.5% and 1.3% of the ED visits.^5^ This is a similar finding to our study where 0.45% of the patients died in the ED. The EDs of tertiary care centers are well-equipped with effective triage systems, specialised staff state-of-the-art diagnostic and therapeutic tools that allow for immediate stabilization and treatment of critically ill patients.^17-19^ This could explain the low death rates of the patients in the ED, as the patients are either stabilized and discharged or they are admitted to other wards of the hospital.^20^ A large volume of patients present to the ED daily, it is said to be one of the most stress- inducing departments to work in the hospital.^21^ Despite the large surge of patients in the ED, the admission rate in our study was only 23.87%. This could be explained by another study which showed that 62% of the patients that visited the ED did not have any urgent issues.^22^ The patients who visit the ED with non-urgent conditions are symptomatically managed and are thus discharged without the need for admission.

Conducting the study within a single tertiary care center in Kathmandu limits its external validity, making it difficult to generalize the findings to other hospitals or regions with distinct patient demographics.^23^ Since this is a cross-sectional study, the data represents only a single point in time, which restricts the ability to observe trends and changes that could be identified through longitudinal research.^24^ The use of convenience sampling may further diminish the generalizability and validity of the results, potentially making it challenging for future studies to replicate the findings due to the non-random sampling approach.^25^ Accurately collecting data on admission rates, patient characteristics, and reasons for ED visits is also difficult, as it may be influenced by inconsistent documentation practices and missing information. Additionally, factors such as seasonal variations, public health campaigns, or changes in healthcare policies could influence ED admission rates, and these elements may not be fully considered in this study.

## CONCLUSIONS

The prevalence of admission of patients visiting the ER was higher than similar US based studies. There was a higher percentage of female patients presenting to the ER, while those being admitted were mostly males.
